# Iron tolerance in rice: an efficient method for performing quick early genotype screening

**DOI:** 10.1186/s13104-019-4362-5

**Published:** 2019-06-25

**Authors:** Adriana Pires Soares Bresolin, Railson Schreinert dos Santos, Roberto Carlos Doring Wolter, Rogério Oliveira de Sousa, Luciano Carlos da Maia, Antonio Costa de Oliveira

**Affiliations:** 1 Universidade Federal do Pampa (UNIPAMPA), Itaqui, RS Brazil; 2 Plant Genomics and Breeding Center, Universidade Federal de Pelotas, Pelotas, RS Brazil; 3 Department of Soils, Universidade Federal de Pelotas, Pelotas, RS Brazil

**Keywords:** Lowland rice, Iron toxicity, Hydroponic culture, Efficient method

## Abstract

**Objectives:**

This study was conducted to establish a method for early, quick and cheap screening of iron excess tolerance in rice (*Oryza sativa* L.) cultivars.

**Results:**

Based on the experiments, iron excess leads to reduction in shoot length (SL) and this can be a useful characteristic for adequate screening of tolerant genotypes. The sensitive genotypes Nipponbare and BR-IRGA 409 indicated higher accumulation of iron in their tissues while BRS-Agrisul and Epagri 108 also accumulated iron, but at lower concentrations. BR-IRGA 410 displayed an intermediate phenotype regarding iron accumulation. No changes in shoot Cu content can be observed when comparing treatments. On the other hand, an increase was seen for Zn and Mn when shoots are subjected to Fe^2+^ excess. Fe stress at a lower concentration than 7 mM increased Zn but decreased Mn contents in shoots of BR-IRGA 409. Strong positive correlations were found here for Fe × Zn (0.93); Fe × Mn (0.97) and Zn × Mn (0.92), probably due to the Fe-induced activation of bivalent cation transporters. Results show that genotypes scored as sensitive present higher concentration of Fe in shoots and this is an efficient method to characterize rice cultivars regarding iron response.

**Electronic supplementary material:**

The online version of this article (10.1186/s13104-019-4362-5) contains supplementary material, which is available to authorized users.

## Introduction

Rice (*Oryza sativa* L.) is an important cereal used to feed more than two-thirds of the worlds population, being the source of more than 20% of the calories consumed by humankind [1]. In this scenario, Brazil, where rice cultivation represents an important economic activity, is the largest rice producer in the Western hemisphere [2].

One of the major abiotic stresses that affect irrigated rice production and expansion is iron toxicity. Iron (Fe) is an essential nutrient for plant metabolic processes such as respiration and photosynthesis. However, when in excess, it becomes a highly toxic element [3–5]. Even though most world’s rice production comes from flood-irrigated farms, flooded soils constitute a hypoxic condition which favors the reduction of iron, increasing the concentration of Fe^2+^ in solution [5, 6]. Iron excess can cause rusty leaf spots, stained leaf edges, reduction of plant growth, tillering and spikelet fertility. Also, reductions in root system development are observed, which can present dark brown color and stunted growth, with few thick roots. In severe cases, these symptoms associate with yield losses up to 100% [7, 8].

Rice genotypes greatly vary in their response to iron toxicity and the use of tolerant cultivars is one of the effective strategies for preventing yield loss, especially for farmers with low income [8, 9].

Considering such background, in this report we aim to evaluate the efficiency of an early, quick and easy method for detection of iron excess tolerance using different rice cultivars.

## Main text

Four Brazilian lowland rice (*Oryza sativa* L.) genotypes were used in this study. These varieties are recommended by the Southern Brazilian Society of Irrigated Rice and are known to be tolerant to iron toxicity by field experiment results [10]: BRS-Agrisul (tolerant), Epagri 108 (tolerant), BR-IRGA 410 (sensitive) and BR-IRGA 409 (sensitive). Nipponbare, the Japanese variety used for the first rice genome sequencing project, is reported as sensitive to iron toxicity. Here Nipponbare was used due to its available molecular data and as a reference for comparisons between different studies [11, 12].

Seeds were disinfected with 20% sodium hypochlorite for 10 min, rinsed for three times in ultrapure water and placed in germination paper for 72 h (25 °C; 16 h of photoperiod; relative humidity of 100%).

Iron stress was performed through the modification of early reports [6]. Seedlings presenting uniform root length were placed in nylon nets fixed on top of plastic pots (2 L), containing modified nutrient [13]: 40 mg L^−1^ of (NH_4_)_2_.SO_4_; 10 mg L^−1^ of KH_2_PO_4_; 40 mg L^−1^ of KNO_3_; 40 mg L^−1^ CaNO_3_; 40 mg L^−1^ of MgSO_4_·7H_2_O; 0.5 mg L^−1^ of MnSO_4_·H_2_O; 0.05 mg L^−1^ of Na_2_MoO_4_·2H_2_O; 0.58 mg L^−1^ of NaCl; 0.2 mg L^−1^ of H_3_BO_3_; 0.01 mg L^−1^ of ZnSO_4_·7H_2_O, 0.01 mg L^−1^ of CuSO_4_·5H_2_O and 2 mg L^−1^ of FeSO_4_·7H_2_O. Seedlings were kept at 25 °C, 16 h of photoperiod for 28 days, with changing the solution every 7 days.

After this period, the seedlings were subjected to different treatments: Control (T1) with standard nutrient solution (2 mg L^−1^ of FeSO_4_·7H_2_O with pH 4.0 ± 0.1); iron excess (T2) with modified nutrient solution (2000 mg L^−1^ of FeSO_4_.7H_2_O with pH 4.0 ± 0.1). Seedlings were kept under these conditions for 3 days. The visual evaluations were performed following the standard evaluation system for rice.

**Fig. 1:**
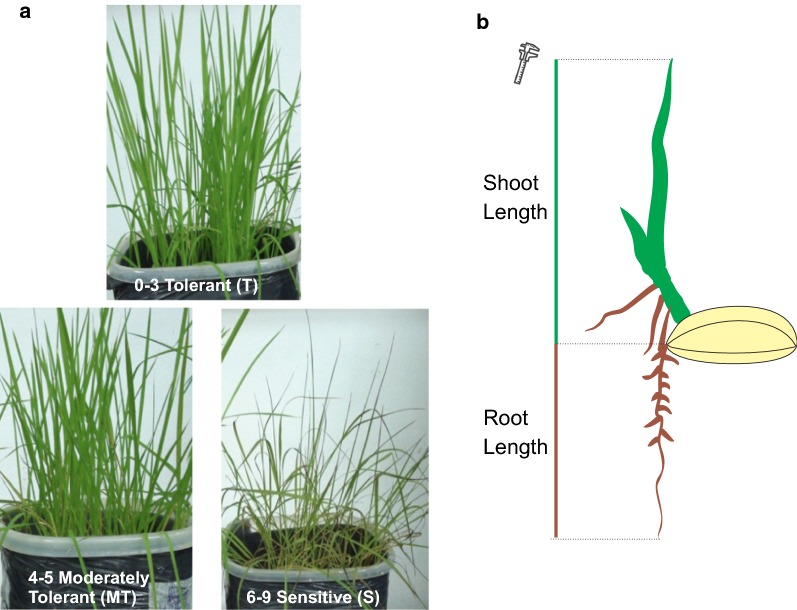
Information about the evaluation procedures on **a** visual symptoms for scoring and **b** shoot and root length measurements

The visual symptoms were based in leaf death and symptom intensity, compared to control (Fig. 1a). The grades ranged from 0 to 9. Tolerant (T) plants received grades 0–3, moderately tolerant (MT) 4–5 and the sensitive (S) 6–9 [14]. After the treatment, root (RL) and shoot (SL) lengths were measured (Fig. 1b). Copper (Cu), zinc (Zn), manganese (Mn) and iron (Fe) contents accumulated in shoots were also evaluated [15].

A completely randomized design in a double factorial 2 × 5 (treatment × genotype) scheme with three replications, where the observation unit consisted on 20 plants per genotype. The data was subjected to analysis of variance (ANOVA) and then to Tukey HSD test and a Pearson’s correlation, both with p ≤ 0.05. Path analysis was performed as described [16, 17]. Data from path analysis are not completely shown here, but most important results are described.

Visual symptoms observed on plants subjected to iron excess toxicity were yellowing, brown spots along the leaves and leaf tip necrosis. The scores based on genotype performance under excess iron are shown on Table 1.

**Table 1:** Leaf bronzing score (LBS) of five genotypes/varieties at 3 days in hydroponic culture under iron toxicity condition

Genotype	LBS1	LBS2	LBS3	Classification
Epagri 108	2	1	2	T
BRS-Agrisul	2	4	3	T
BR-IRGA 410	5	4	6	MT
Nipponbare	7	8	8	S
BR-IRGA 409	6	7	7	S

The LBS numbers follow a scale from 0 to 9, adapted from standard evaluation system for Rice used by IRRI

Classification regarding iron tolerance levels, T (tolerant 0–3), MT (moderately tolerant 4–5) and S (sensitive 6–9)

Epagri 108 (tolerant in field conditions) barely presented Fe^2+^ toxicity symptoms while the genotype BR-IRGA 409 (sensitive in field conditions) presented easily identifiable Fe^2+^ toxicity symptoms under iron excess conditions (T2). These results agree with field assays [10]. Nipponbare presented higher scores, being ranked as sensitive to iron stress, classification in agreement with previous reports obtained in hydroponic systems [12]. The use of bronzing scores, measured in the field or in hydroponic systems, has shown to be efficient on the discrimination of tolerant genotypes, being associated to grain yield [8, 9, 18]. However, since in early developmental stages changes in SL, RL and nutrient accumulation in tissues have been reported to constitute an objective form of evaluation that can be used in conjunction with bronzing scores, these were also evaluated during this study [19–21].

**Fig. 2:**
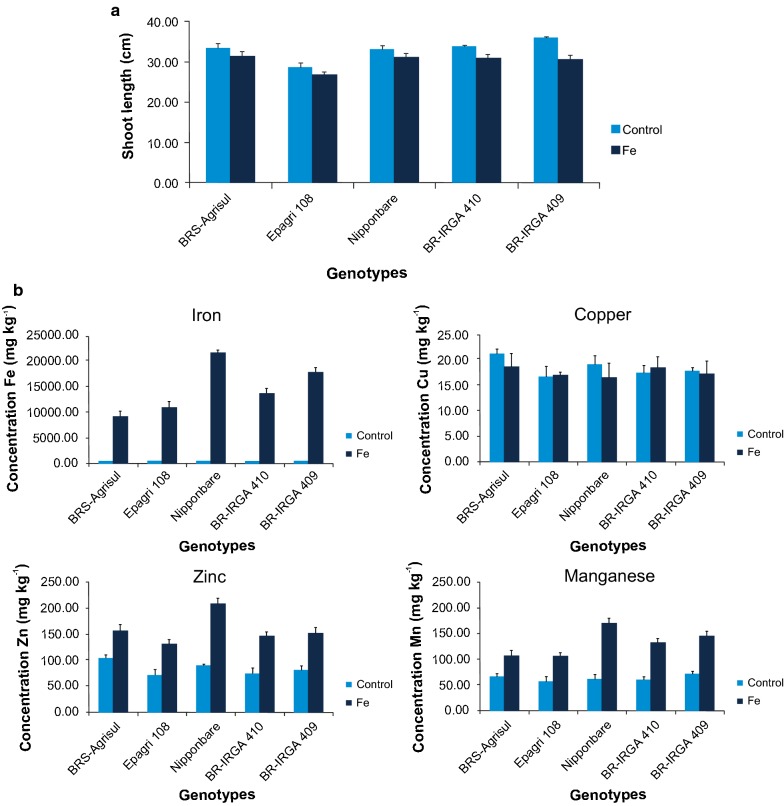
**a** Shoot length (SL) of each genotype, of the five lowland rice genotypes subjected to standard conditions (control) and to iron excess; **b** micronutrient content in shoots of five lowland rice genotypes subjected to standard conditions (control) and to iron excess. A graph showing the relative performance of these cultivars and Tukey’s pairwise comparisons is available in the Additional file 1

It is shown here that iron excess can lead to reduction in SL (Fig. 2a) and, as other previous studies suggest, this can be a useful characteristic for helping the screening of tolerant genotypes [19, 21].

The sensitive genotypes Nipponbare and BR-IRGA 409 indicated higher accumulation of iron in their tissues (Fig. 2b), while BRS-Agrisul and Epagri 108 (both previously characterized as tolerant) also accumulated iron, but at lower concentrations (i.e., ca. 50% less). It is shown that BR-IRGA 410 display an intermediate phenotype regarding iron accumulation.

It is also shown that BRS-Agrisul (a medium cycle genotype; 121–130 days) accumulated lower amounts of iron than other medium cycle genotypes such as BR-IRGA 410 and BR-IRGA 409, showing that the time from germination to grain production is not the cause of differences in the amount of iron accumulated in tissues.

No changes in shoot Cu content can be observed when comparing treatments (Fig. 2b). On the other hand, an increase was seen for Zn and Mn (Fig. 2b) when shoots are subjected to Fe^2+^ excess (T2). Fe stress at a lower concentration than 7 mM increased Zn but decreased Mn contents in shoots of BR-IRGA 409 [22].

Strong positive correlations were found here for Fe × Zn (0.93); Fe × Mn (0.97) and Zn × Mn (0.92) (Table 2), probably due to the Fe-induced activation of bivalent cation transporters [23].

Iron content is negatively correlated with SL (− 0.37) and RL (− 0.42), highlighting the impact that excessive accumulation of this metal has on rice growth and development (Table 2). Although similar results are observed for correlations between Mn and Zn with SL and RL, these are only significant for RL (Table 2) and, according to the path analysis (data not shown), it seems to be an indirect effect of iron accumulation.

**Table 2:** Pearson’s correlation coefficient within and between traits (SL and RL) and micronutrients (iron, copper, zinc and manganese) for five genotypes/varieties under control and iron toxicity conditions in hydroponic culture

Variables	Fe	Cu	Zn	Mn	SL	RL
Fe	1	− 0.19	0.93*	0.97*	− 0.37*	− 0.42*
Cu		1	− 0.02	− 0.19	0.24	0.26
Zn			1	0.92*	− 0.27	− 0.38*
Mn				1	− 0.30	− 0.49*
SL					1	0.08
RL						1

* Significant at p ≤ 0.05

This quick and easy modification of the protocol described by [6] proved to be an efficient method to select tolerant Brazilian lowland rice genotypes for iron excess tolerance. Plants showing higher Fe^2+^ accumulation in shoots (BR-IRGA 409 and Nipponbare) were the same identified as sensitive to Fe^2+^ by the bronzing score. Besides, the genotype BR-IRGA 409, characterized as sensitive, is the one showing the highest reduction of SL due to iron toxicity. BR-IRGA 410, an intermediate phenotype for shoot Fe^2+^ accumulation, is characterized as moderately tolerant in symptom score evaluation. BRS-Agrisul and Epagri 108 which are the genotypes displaying the lowest Fe^2+^ accumulation levels in shoots, are characterized as tolerant in visual symptom evaluation.

In Brazil the search for iron tolerant genotypes has been performed for many years. The methods used involve field tests during different years/growing seasons [24]. Even today, this is the most acceptable method, being necessary to have credibility when registering a cultivar. However, for a quick and inexpensive initial selection in breeding programs, efficient protocols to predict genotype performance have not yet been achieved [25]. Different methods have been tested, these include pot- or tank-based screening procedures that have been presented over time until very recently [26–28].

It is common for the methods tested to find an adequate correlation with the field experiments using soils of the site of interest [29]. The method presented here is useful for an initial selection of genotypes, without considering the soil, since the removal of this from its place of origin does not guarantee perfect reproducibility of the results, either by the modification of the structure or by the lack of local climatic elements. Thus, a prior soil-independent evaluation can be useful to reduce the number of genotypes to be tested and the cost, taking only the most promising ones to field.

## Limitations

The limitations of this work are the use of just five genotypes. Although they are contrasting for iron response and should be sufficient to explain the responses, it could be considered a limitation.

## Additional files

**Additional file 1: Figure S1.** Relative performance obtained by the division of the values of plants under stress by the values of the control treatment. Columns followed by the same letters do not differ significantly (Tukey’s pairwise comparisons, p < 0.05).

## Data Availability

The datasets used and/or analysed during the current study are available from the corresponding author on reasonable request.

## Abbreviations

ANOVA: analysis of variance
MT: moderately tolerant
RL: root length
S: sensitive
SL: shoot length
T: tolerant
Tukey HSD: Tukey honest significant difference
