# Supplementary material for: Iron tolerance in rice: an efficient method for performing quick early genotype screening
Source: BMC Res Notes. 2019 Jun 25;12:361. doi: 10.1186/s13104-019-4362-5 (PMC6593560; doi:10.1186/s13104-019-4362-5)
Supplement: Supplementary file 1 — Additional file 1: Figure S1. Relative performance obtained by the division of the values of plants under stress by the values of the control treatment. Columns followed by the same letters do not differ significantly (Tukey’s pairwise comparisons, p < 0.05). [file 13104_2019_4362_MOESM1_ESM.pdf]

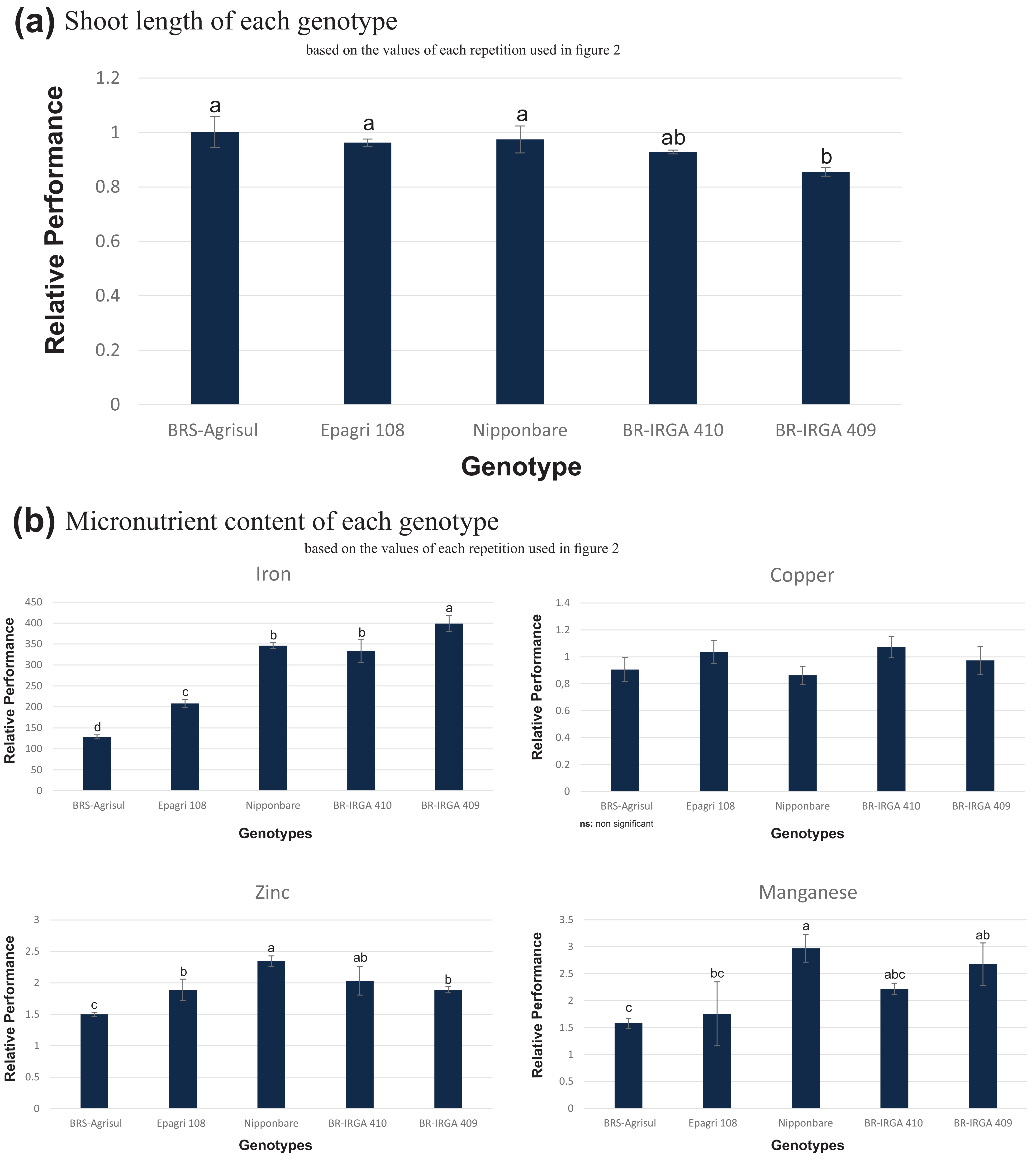

Figure S1. Relative performance obtained by the division of the values of plants under stress by the values of the control treatment. Columns followed by the same letters do not differ significantly (Tukey's pairwise comparisons, $p < 0.05$).
